# Determining the meter of classical Arabic poetry using deep learning: a performance analysis

**DOI:** 10.3389/frai.2025.1523336

**Published:** 2025-02-14

**Authors:** A. M. Mutawa, Ayshah Alrumaih

**Affiliations:** ^1^Department of Computer Engineering, College of Engineering and Petroleum, Kuwait University, Safat, Kuwait; ^2^Department of Computer Sciences, University of Hamburg, Hamburg, Germany

**Keywords:** Arabic poetry, Arabic meters, Bi-LSTM, deep learning, machine learning, natural language processing

## Abstract

The metrical structure of classical Arabic poetry, deeply rooted in its rich literary heritage, is governed by 16 distinct meters, making its analysis both a linguistic and computational challenge. In this study, a deep learning-based approach was developed to accurately determine the meter of Arabic poetry using TensorFlow and a large dataset. Character-level encoding was employed to convert text into integers, enabling the classification of both full-verse and half-verse data. In particular, the data were evaluated without removing diacritics, preserving critical linguistic features. A train–test–split method with a 70–15–15 division was utilized, with 15% of the total dataset reserved as unseen test data for evaluation across all models. Multiple deep learning architectures, including long short-term memory (LSTM), gated recurrent units (GRU), and bidirectional long short-term memory (Bi-LSTM), were tested. Among these, the bidirectional long short-term memory model achieved the highest accuracy, with 97.53% for full-verse and 95.23% for half-verse data. This study introduces an effective framework for Arabic meter classification, contributing significantly to the application of artificial intelligence in natural language processing and text analytics.

## 1 Introduction

Arabic prosody (Arud) has been studied for many years in morphology and phonetics. The study of meters in poetry enables us to determine whether the poetry is sound or broken (Jones, 2011). Some of the terminology used most frequently in Arabic prosody are as follows: a single line of the poetry comprises two verses, each half-verse called a “bayt.” The first verse is “sadder,” and the second is “ajuz.” Classical Arabic poetry, defined by units called meters, was analyzed by the famous lexicographer and grammarian Al-Khalil ibn Ahmad al-Farahidi in the eighth century (Alnagdawi et al., 2013). The meter is based on the syllables in a word and consists of two parts: short and long syllables. The 16 meters are Tawil, Basiit, Madid, Wafir, Kamil, Hazaj, Rajaz, Ramal, Munsarih, Khafif, Muqtadab, Mujtath, Mudari’, Sarii’, Mutaqarib, and Mutadarik. The ode may consist of 120 lines, split into two half-lines characterized by their meters, repeated for the whole verse. Al-Farahidi represented some feet provided in a rhythmic to make it easy to remember the meter (fa’uulun, mafaa’iilun).

Poetry is a way of communication and interaction and an essential aspect of any language and literature. Communities, nations, and societies have expressed themselves through poetry for ages (Lavzheh, 2009). Poetry is hard to understand as it has a specific pattern and underlying meanings in its words and phrases, making it different from prose. It is necessary to understand the structure to understand the poetry completely. Bahar is the meters in Arud science. Arud science helps divide Arabic poems into 16 meters, making them easy to understand without referring to the context (Alnagdawi et al., 2013). Classical Arabic poetry can be recognized and understood using various methods and tools. Arud is the rule and regulations of poems used in many languages (Abuata and Al-Omari, 2018). Poetry is different from prose, mainly because of its form and structure. Poetry consists of tone, metrical forms, rhythm, imagery, and symbolism. In Arabic poetry, each line ends with a specific tone. The field that studies rhyme and rhythm is called prosody and is complex due to many overlapping rules (Khalaf et al., 2009).

There are two vowels in modern and classical Arabic: long and short. The long vowels are explicitly written, and short vowels are also called diacritic. Various attempts have been carried out to implement Arabic text. A proposal was made to use Arabic diacritics or ‘harakat’ for text hiding for security purposes (Ahmadoh and Gutub, 2015). The diacritics in Arabic are split into three parts as shown in Table 1. The majorty of studies in this field use a deep learning method to diacritize the Arabic text before loading it into the model (Abandah et al., 2022; Abandah et al., 2020; Kharsa et al., 2024).

**Table 1:** Arabic diacritic types.

Diacritic	Types	Example
Harakat	“fatha” “dahmmah” “kasrah” “sukon”	شَرِب الطفْلُ الحليبَ
Tanween	Tanween fateh, tanween dham and tanween kasr	بارداً, باردٌ, باردٍ
Dhawabet	Shad, mad	الشَّمس, آية

Artificial intelligence (AI) has become exponentially more practical and significant over the last few years. The AI-enabled state-of-the-art technologies have expanded substantially and shown effective results in almost every industry, such as security (Wu et al., 2020), surveillance, health (Davenport and Kalakota, 2019), automobiles (Manoharan, 2019), fitness tracking (Fietkiewicz and Ilhan, 2020), and smart homes (Gochoo et al., 2021). In general, AI and machine learning (ML) are correlated. They are primarily used to develop intelligent systems (Das et al., 2015). Deep learning (DL) is a type of ML that allows computers to learn from data representation with more neural levels. Convolutional neural networks (CNN) have revolutionized image, video, and audio processing, and recurrent neural networks (RNN) have gained insight into text and speech sequential data (LeCun et al., 2015). The design of any deep learning model must consider the choice of algorithm. Most sequential applications follow the RNN model (Iqbal and Qureshi, 2022), and it has the context of previous input but not the future context of the speech or text data. Bidirectional recurrent neural networks (Bi-RNN) extract the context of data in both forward and backward directions (Schuster and Paliwal, 1997).

The proposed research offers substantial contributions to text analytics and natural language processing (NLP), particularly focusing on the complex issue of classifying Arabic poetry meters. This study employed Arabic text without removing diacritics from the poetry dataset. The 14 meters of the Arabic poem were considered. Two meters were removed because of very little data compared to other meters. The RNN models such as long short-term memory (LSTM), gated recurrent units (GRU), and Bi-RNN models, such as bidirectional LSTM (Bi-LSTM), are used to implement the proposed study. Despite the long history of Arabic poetry, automated techniques for meter classification have not received much attention. The proposed study utilized a large dataset and advanced neural network models. The main contribution of the study is defined as follows:

- Development of a DL framework utilizing TensorFlow for the categorization of Arabic poetry meter. The framework is specifically designed to categorize Arabic poetry meters, a field that presents linguistic and structural difficulties because of the complexity and variety of the Arabic language.
- Employing character-level encoding to transform text into integers for efficient categorization. This encoding enables the model to discern complex language patterns and nuanced differences at the character level, facilitating more efficient classification.
- To strengthen the robustness and usefulness of the classification methodology, the study employed both full-verse and half-verse types of Arabic poetry. This analysis allows the model to accurately identify poetry of diverse lengths and structural complexities, offering a thorough comprehension of Arabic poetic traditions.
- The research conducts an extensive assessment of several DL architectures, including LSTM, GRU, and Bi-LSTM, to determine the most efficient model for Arabic meter categorization. The Bi-LSTM model exhibited exceptional performance, attaining the greatest classification accuracy and highlighting its proficiency in managing the sequential and contextual intricacies of Arabic poetry.
- The findings of the study highlight the efficacy of DL techniques in tackling the complex nature of Arabic poetry meter classification. The research utilizes neural architectures and encoding methodologies to provide useful insights into the adaptation of existing NLP methods for the linguistically rich and morphologically complicated Arabic language.

The remaining section of this paper is organized into five sections. Section 2 explains the literature review, including Arabic meter and DL models. Section 3 describes the methodology used and the model algorithm. Section 4 presents the results in detail, with a discussion in section 5. Section 6 describes the conclusion with future study.

## 2 Literature review

Alnagdawi et al. (2013) used another tool for language recognition to find the meter of Arabic poems. This tool works in three steps: first, it converts poetry into Arud form. The second step is the segmentation of the Arud form. In this phase, the Arud state is divided into sounds, such as short sounds, vowel or long sounds, and consonants. The sound string was sent to the final stage at the end of the second step, and the poetry meter was detected. It is compared with grammar to check its validity. If the grammar is valid, the verse belongs to 16 meters. The meter patterns match the poem’s words, identifying the meter’s name.

A considerable body of literature is on recognizing Arabic poetry using deep learning algorithms. Baïna and Moutassaref (2020) developed an algorithm that accurately identifies the meter of the poem and outputs the ‘Arud’ writing in addition to the meter. The algorithm follows five phases. First, it adds diacritics to the verse. This step is significant as it might impede moving to the next step. Second, it transforms the diacritics into ‘Arud’ writing. Third, it utilizes binary representation to convert the ‘Arud’ writing, where 1 represents a ‘haraka’ and 0 illustrates a ‘sukon.’ Fourth, the algorithm identifies the meter based on the binary representation. The fifth and final step includes detecting the errors and ensuring the meter matches the poem.

Furthermore, Albaddawi and Abandah (2021) proposed a narrow, deep neural network with significantly high accuracy. The proposed network consists of an embedding layer at its input, five Bi-LSTM layers, a concentration layer, and an output layer with softmax activation. Similarly, Abandah et al. (2020) suggested improving the recognition of diacritics via a specific neural network. This strategy tries to enhance readability and recognition accuracy. Moreover, identifying the meter of an Arabic poem may be a long and complicated process that involves a few steps (Al-shaibani et al., 2020). A study by Ahmed et al. (2019) utilized ML algorithms to identify and classify Arabic texts. The study supports linear vector classification and naïve Bayes classification, which showed the highest precision. Many studies have been conducted on analyzing Arabic poetry. Formulating one system or technique to identify meters in Arabic poetry is challenging. A study on identifying Arabic poetic meter (Saleh and Elshafei, 2012) suggested a method that produces coded Al-Khalili transcriptions of Arabic.

Abuata and Al-Omari (2018) electronically analyzed the Arud meter of Arabic poetry. They introduced an algorithm to determine the meter of Arud for any Arabic poetry. The algorithm works on well-defined rules applied only to the first part of the poem verse. Moreover, some of the most outstanding works in Arabic poetry are the computerization of Arabic poetry meters (Khalaf et al., 2009). It focuses on computerizing El-Katib’s method for analyzing Arabic poetry. The linguist El-Katib proposed a study in which poetry is converted into binary bits and given decimal codes. This system was helpful for educational purposes. Many students and teachers use it to understand prosody. The computerized and systematic analysis of prosody also minimizes the chance of error.

Attempts have been made to develop algorithms that recognize modern Arabic poetry meters (Abandah et al., 2022; Abandah et al., 2020; Al-shaibani et al., 2020). For instance, an algorithm has been introduced to identify standard features of classical Arabic poems (Zeyada et al., 2020). These features include rhyme, rhythm, punctuation, and text alignment. This algorithm can only recognize whether the Arabic piece is poetic or non-poetic but cannot acknowledge its meter. Furthermore, an algorithm has been developed to detect the Arabic meter of certain poetry and convert the verse into ‘Arud’ writing (Al-Talabani, 2020). It classifies Arabic poetry using meters or ‘Bahr’ and investigates methods of detecting Arabic poems in rhythm, rhyme, and meter. It utilizes time and non-time series representation of the Mel-frequency cepstral coefficients (MFCC) and linear predictive cepstral coefficients (LPCC) features to recognize automated ‘Arud’ meters. Arabic ‘Arud’ meters seem to possess a time-series nature; however, the non-time series representation performs better.

Another detection method includes a comparison that has been conducted between modern and classical Arabic poetry (Almuhareb et al., 2015). The results reveal that contemporary Arabic poetry lacks more distinctive features than classical poetry. For instance, modern Arabic poetry is characterized by partial meter, the uneven lining of verses, word repetition, usage of punctuation, and irregular rhyming. At the same time, classical Arabic poetry is characterized by a regular rhyme, a single meter, even lining of verses, and self-contained lines. Similarly, Berkani et al. (2020) notes that extracting the meter of the poem using automatic meter detection methods requires challenging data collection and processing efforts. Syllable segmentation and similarity checks are performed. This method has further proven the high accuracy of meter detection. Finally, creating detecting algorithms may considerably improve the efficiency and accuracy of Arabic poetry identification methods.

The LSTM model is one of the most widely used RNN systems for vanishing gradients (Hochreiter and Schmidhuber, 1997). In addition, these networks have several advantages compared to conventional RNN systems, including the ability to sustain prolonged interrelationships and exhibit a stochastic nature when dealing with time-series input data. With RNN or LSTM, the uniform weight is retained across all layers, limiting the number of parameters the network must learn. The LSTM model had more parameters, which made it slower.

Later, GRUs were proposed as a better alternative to LSTMs and have gained significant recognition (Cho et al., 2014). In addition, GRUs have been recognized to be effective in numerous applications using sequential or time-series input (Dey and Salem, 2017). For instance, they have been incorporated in diverse areas such as speech synthesis, NLP, and signal processing. Furthermore, LSTM, RNNs, and GRUs have been exhibited to operate better in long-sequence applications. In GRUs, gating network signaling plays a significant role as it controls how inputs and memory are used to update current activations. Each gate has weights that are adapted and modified in the learning phase. However, these systems enable effective learning in RNNs, increasing parameterization. It leads to a simpler RNN model with a higher computational cost. The LSTM and GRU differ because the former utilizes three novel gate networks, whereas the latter uses only 2.

The Bi-LSTM neural network comprises LSTM units that operate in both directions to exploit contextual information from the past and future (Liang and Zhang, 2016). In addition, with Bi-LSTM, long-term dependencies can be learned without maintaining redundant background information. Thus, it has projected significant performance for sequential modeling issues and is generally used for text classification (Huang et al., 2015; Al-Smadi, 2024). Bi-LSTM networks transmit forward and reverse phases in both directions, unlike LSTM networks, which communicate only in one direction.

Many NLP sequences-to-sequence methods use LSTM, GRU, Bi-LSTM, and Bi-GRU deep learning models (Liang and Zhang, 2016; Wazery et al., 2022; Yin et al., 2017; Huang et al., 2015). In recent years, ML has become a formidable method for text analysis, exhibiting adaptability across several applications. Diverse ML methodologies have been effectively utilized in tasks such as dialect detection, spam detection, poetry classification, text classification, and sentiment analysis (Ahmed et al., 2019; El Rifai et al., 2022; Chen et al., 2022; Abdulghani and Abdullah, 2022; Alqasemi et al., 2021; Zivkovic et al., 2021), demonstrating their proficiency in managing intricate textual data.

An important use of ML is sentiment categorization, employed for the identification of insider threats. Recent studies by Mladenovic et al. (2024) have illustrated that sentiment analysis can be augmented through optimized classifiers, thereby enhancing the precision of threat detection in organizational contexts. In spam email screening, NLP combined with ML has shown success (Bacanin et al., 2022). It explains how swarm intelligence can maximize conventional ML techniques, thereby improving user experience and spam detection accuracy. Another study by Kozakijevic et al. (2024) examined the incorporation of sentiment analysis in e-commerce, highlighting its significance in assessing seller reputation and influencing consumer choices. They attained a maximum accuracy of 88% by integrating transformer embeddings with an efficient extreme gradient boost model, refined via a modified firefly approach.

## 3 Materials and methods

**Figure 1:**
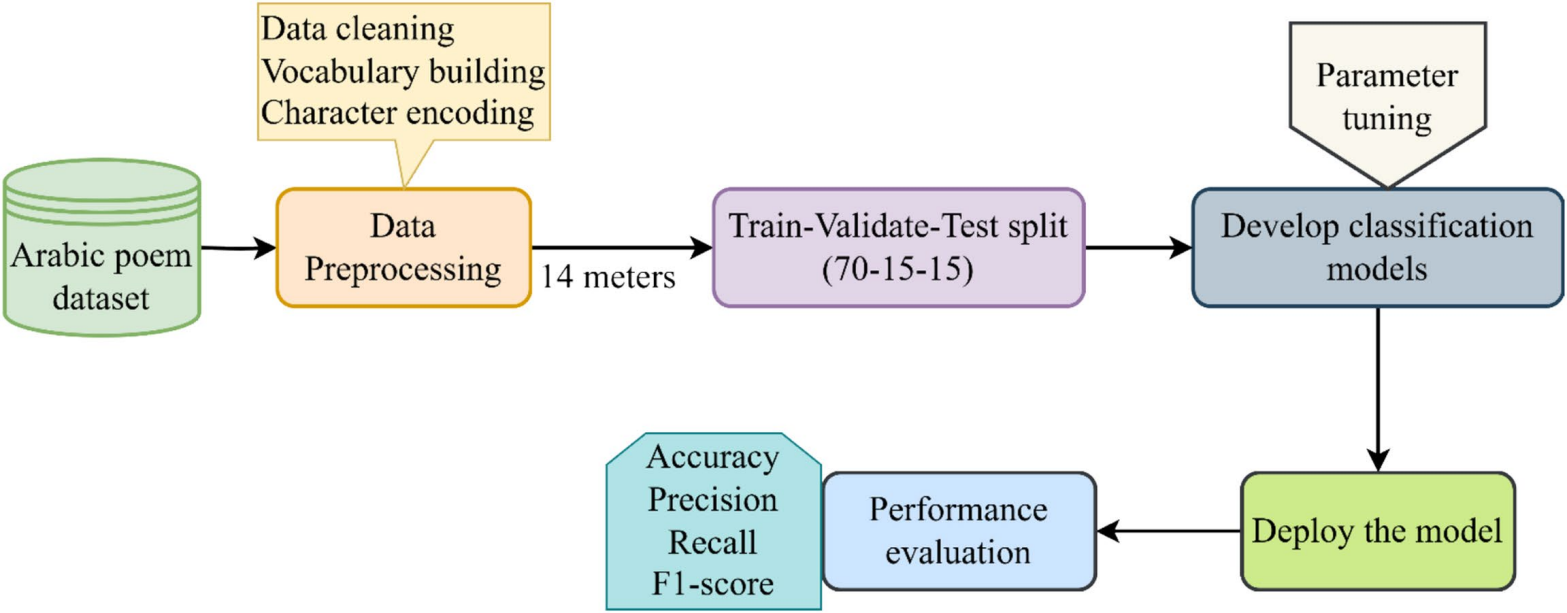
Overview of the research methodology.

The methodology of the study is shown in Figure 1. The key phases of the study include fetching the dataset, preprocessing and splitting the data, and developing and applying the DL models. The results were evaluated using a combination of accuracy, precision, recall, and the F1 score.

### 3.1 Dataset and preprocessing

**Figure 2:**
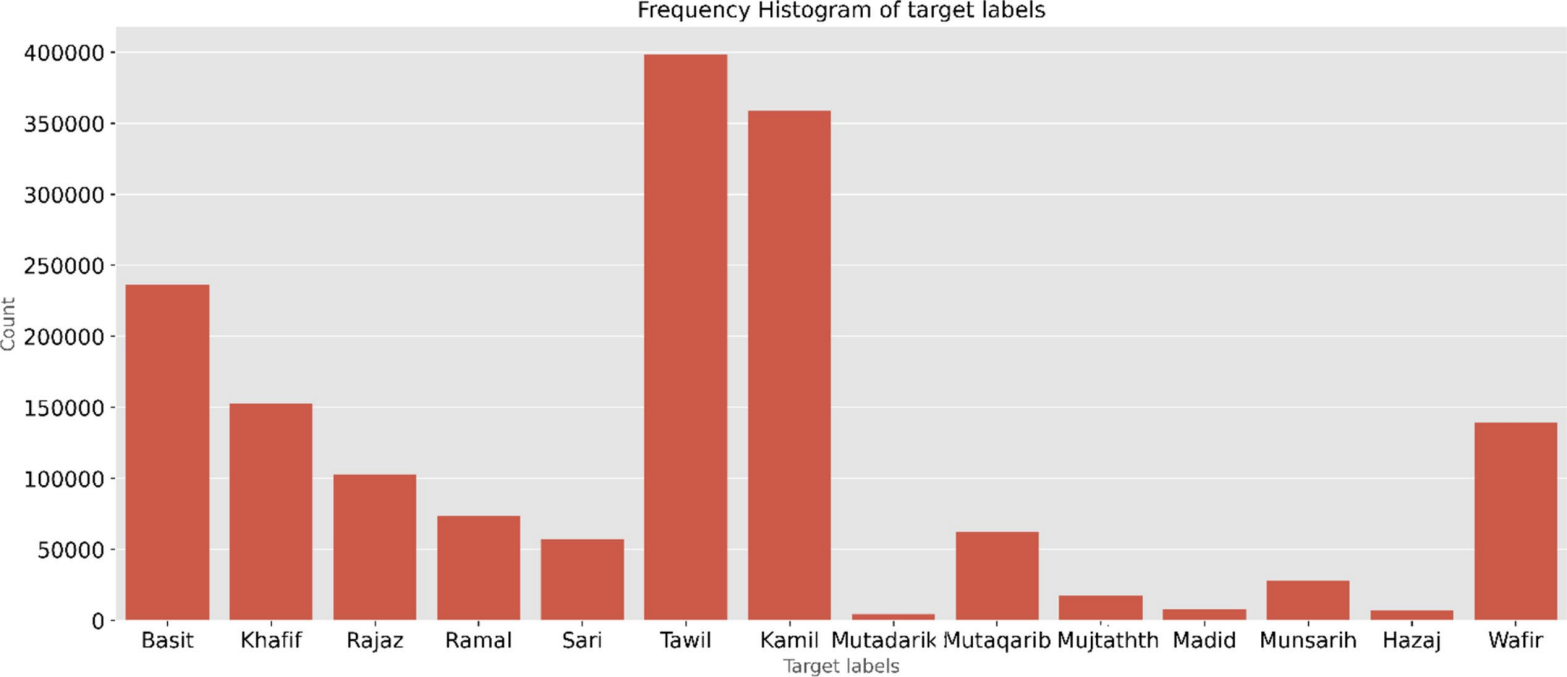
Full-verse count of the 14 meters in the dataset.

The dataset contains 1,862,046 verses with 22 meters (Yousef et al., 2019). The data are in a well-structured format. The central 16 meters consist of a data size of 1,647,854. Two meters with fewer verses are avoided when classifying the meters. After eliminating the empty cells, the total number of verses in the 14 meters of data, which include both right and left verses, is 1,646,771. The count of each meter label with a full-verse is depicted in Figure 2. The minimum count is for the Mutadarik meter, 4,507 verses, and the maximum is for the Tawil meter, 398,239 verses. To address data scarcity for certain meters and improve the robustness of the models, half-verse data were doubled during training by treating the left and right verses of each meter as independent samples.

The dataset underwent a thorough cleaning process to enhance its quality and suitability for deep learning. Non-Arabic characters, symbols, and other irrelevant text artifacts were systematically removed. This step ensured that only meaningful linguistic content remained, aligning the dataset with the methodological requirements. The preprocessing methodology closely follows the approach described in Al-shaibani et al. (2020) including the construction of a character-level vocabulary. The character-level encoding uses the index value for each cleaned text and implements DL models. Parameter tuning was conducted for each deep learning model to optimize performance, with attention to hyperparameters such as learning rate, batch size, and sequence length. The data are split into 70% training and 15% validation; the remaining 15% are set as unseen data for testing.

### 3.2 Deep learning models

This study uses the deep neural network (DNN) architecture. The two main architectures of DNN are RNN and CNN (Yin et al., 2017). LSTM, GRU, and Bi-LSTM are models under RNN (Sherstinsky, 2020). The base model for LSTM consists of four layers. The first layer of the sequential model is the input layer with the size of the padded sequence, which is then given to the embedding layer with the output dimension kept as 64. The embedding layer will learn how to map the characters to vectors. The output from the embedding layer is fed into the LSTM layer with units 256, recurrent, and the activation function is set as the default. The LSTM layer is added accordingly to increase the hidden layers. At this moment, the return sequence parameter should be set as ‘True.’ The GRU model is like the LSTM model. In both models, sentence processing is only in one direction.

**Figure 3:**
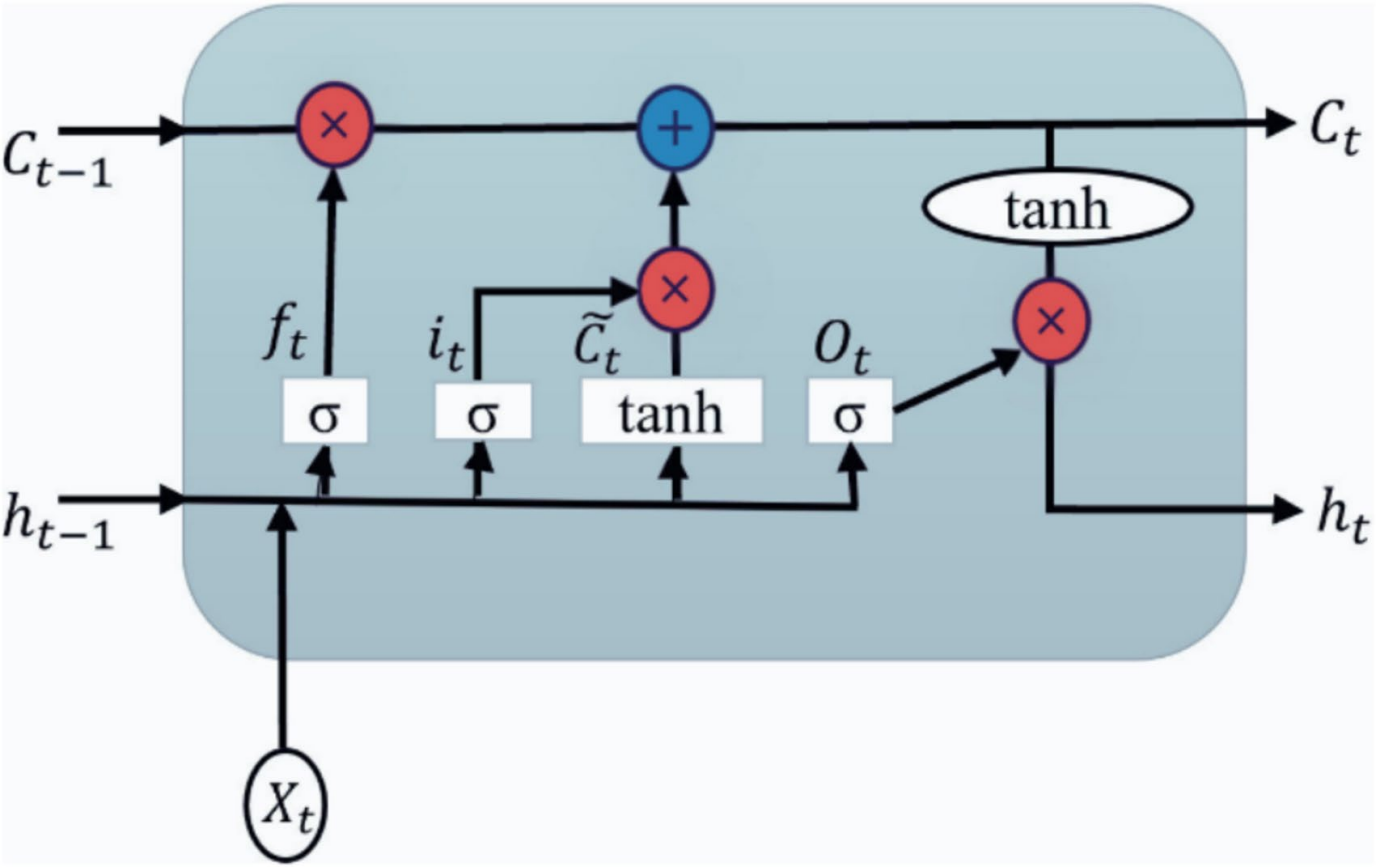
Internal architecture of the LSTM layer.

The LSTM layer is depicted in Figure 3. It allows the model to store the information for future access and has a hidden state: short-term memory. There are three gates for LSTM such as input (i_t_), output (O_t_), and forget gate (f_t_). A time step is indicated by the subscript ‘*t*.’ The LSTM has three inputs: an input vector at the current time stamp (X_t_), a cell or memory state vector (C_t-1_), and a hidden state vector at the previous time stamp (h_t-1_). The symbol ‘×’ denotes the element-wise product or the Hadamard product. $\tilde{C_{t}}$is the cell state activation vector or the candidate memory vector (Harrou et al., 2021).

As a first step, what information the cell state should discard should be determined. It is accomplished by the sigmoid activation function (*σ*) in the forget gate and applies the sigmoid function to the current input vector X_t_ and the past hidden state vector h_t-1_ as shown in Equation 1. Input activations activate memory cells through input gates.


(1) $$f_{t}=\sigma \left(w_{f}X_{t}+u_{f}h_{t-1}+b_{f}\right)$$

where f_t_ = forget gate, w_f_ and u_f_ are the weight matrices of the forget gate, X_t_ is the actual input, b_f_ is the bias vector, h_t-1_ is the hidden state output from the previous time stamp, and *σ* is the sigmoid activation function. The result from Equation 1 is in the range of 0 and 1. The element-wise product of C_t-1_ and f_t_ decides what information to retain and forget.

The second step is to update the memory cell with an input gate as shown in Equation 2. The sigmoid function indicates two values: if it is 1, the actual data are unchanged, and if it is 0, it will be dropped. A tanh function is applied to the selected input values, which indicates a range from −1 to +1. It creates a new vector of values, a candidate memory cell (Equation 3).


(2) $$i_{t}=\sigma \left(w_{i}X_{t}+u_{i}h_{t-1}+b_{i}\right)$$

where i_t_ = input gate, w_i_ and u_i_ are the weight matrices of the input gate, b_i_ is the bias vector, X_t_ is the actual input, h_t-1_ is the hidden state output from the previous time stamp, and *σ* is the activation function.


(3) $$\tilde{C_{t}}=\sigma \left(w_{c}X_{t}+u_{c}h_{t-1}+b_{c}\right)$$

where $\tilde{C_{t}}$= candidate memory cell, w_c_ and u_c_ are the weight matrices, b_c_ is the bias vector, X_t_ is the actual input, h_t-1_ is the hidden state output from the previous time stamp, and σ is the activation function.

The following step involves updating and converting the previous cell state C_t-1_ to the new C_t_. Equation 4 is defined as:


(4) $$C_{t}=f_{t}\cdot C_{t-1}+i_{t}\cdot \tilde{C_{t}}$$

where f_t_ = forget gate calculated from Equation 1, C_t-1_ is the memory state vector of the previous time stamp, i_t_ = input gate calculated from Equation 2, and $\tilde{C_{t}}$is the candidate memory cell from Equation 3.

The final stage is to decide what portion of the output will be selected. It is done in two steps. First, the sigmoid function is performed with the input to determine the quantity of cell state to transmit as the output (Equation 5). The tanh operation is then applied to the new cell state C_t_, and the sigmoid result is multiplied by the result (Equation 6). Thus, the outcome is based only on the selected portions.


(5) $$O_{t}=\sigma \left(w_{o}X_{t}+u_{o}h_{t-1}+b_{o}\right)$$

where O_t_ = output gate, w_o_ and u_o_ are the weight matrices of the output gate, b_o_ is the bias vector, X_t_ is the actual input, h_t-1_ is the hidden state output from the previous time stamp, and *σ* is the activation function.


(6) $$h_{t}=\tanh\left(C_{t}\right)\cdot O_{t}$$

where O_t_ = output gate calculated from Equation 5, and new cell state C_t_ calculated from Equation 4.

**Figure 4:**
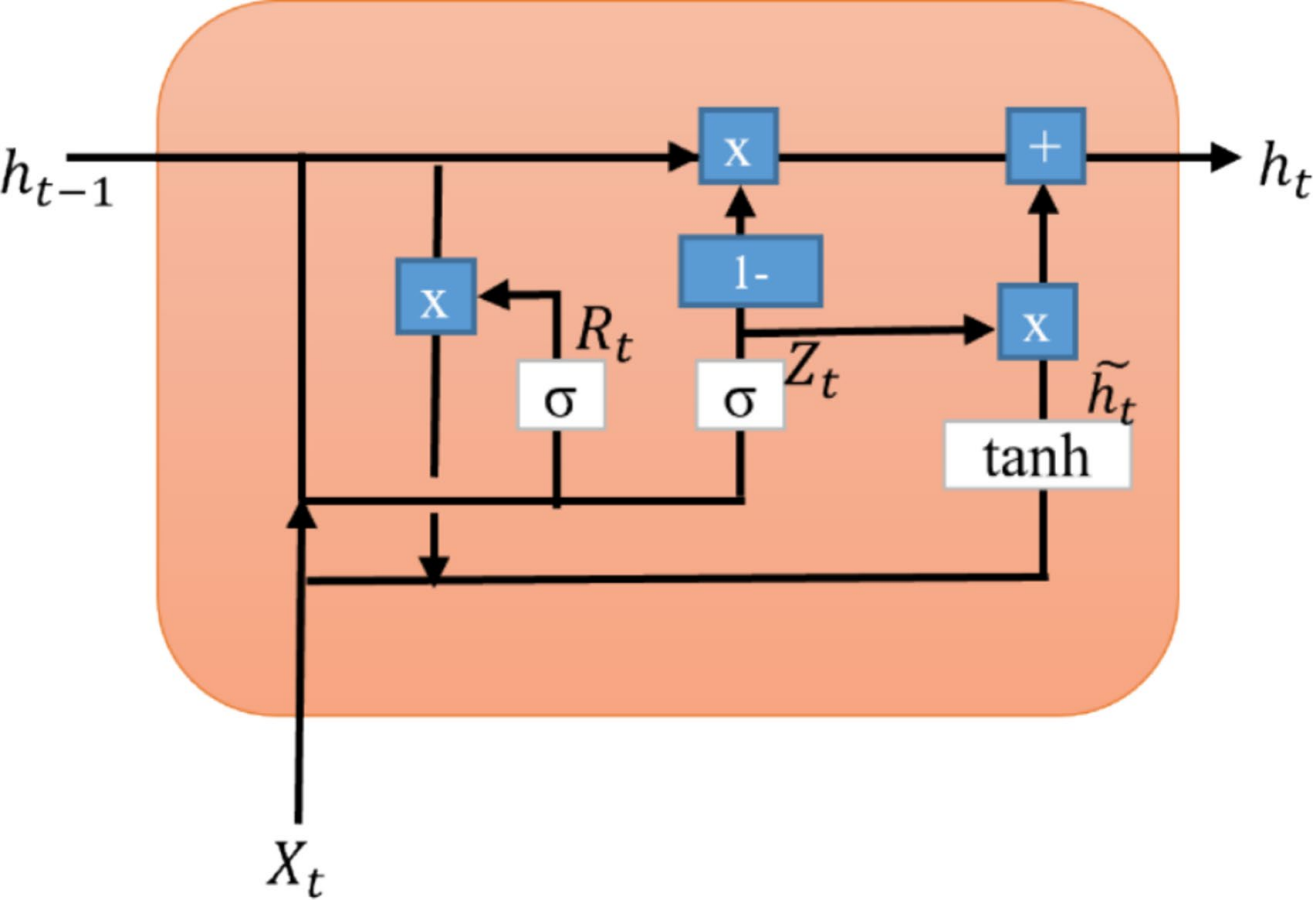
Internal structure of GRU layer.

The GRU layer is illustrated in Figure 4. A reset gate and an update gate are two gates. However, the GRU requires fewer parameters to train than the LSTM model, which runs faster. The reset gate (R_t_) regulates the amount of the initial state that needs to be remembered. Similarly, an update gate (Z_t_) enables us to assess how much the new form replicates the previous one. As each hidden unit reads/generates a sequence, these two gates control how much of it is remembered or forgotten (Harrou et al., 2021).

The reset gate performs similar functions to the forgotten gate of LSTM (Equation 7). It manages the short-term memory of the network. A decision is made regarding what information should be forgotten.


(7) $$R_{t}=\sigma \left(w_{r}X_{t}+u_{r}h_{t-1}+b_{r}\right)$$

where R_t_ = reset gate, w_r_ and u_r_ are the weight matrices of the reset gate, b_r_ is the bias vector, X_t_ is the actual input, and h_t-1_ is the hidden state output from the previous time stamp.

The update gate manages the long-term memory of the network. It accomplishes a similar task as the forget and input gates of an LSTM. It determines what data should be removed and what new data should be added (Equation 8).


(8) $$Z_{t}=\sigma \left(w_{z}X_{t}+u_{z}h_{t-1}+b_{z}\right)$$

where Z_t_ = update gate, w_z_ and u_z_ are the weight matrices of the update gate, b_z_ is the bias vector, X_t_ is the actual input, and h_t-1_ is the hidden state output from the previous time stamp.

The hidden state ($\tilde{h_{t}}$) of the candidate is also called an intermediate memory unit, which combines the previously hidden state vector in the reset gate with the input vector (Equation 9).


(9) $$\tilde{h_{t}}=\tanh\left(w_{h}X_{t}+u_{h}\left(R_{t}\cdot h_{t-1}\right)+b_{h}\right)$$

where $\tilde{h_{t}}$ = candidate hidden state vector, w_h_ and u_h_ are the weight matrices, b_h_ is the bias vector, R_t_ = reset gate calculated from Equation 7, X_t_ is the actual input, and h_t-1_ is the hidden state output from the previous time stamp.

The final hidden state is determined based on the update gate and candidate hidden state. The update gate is multiplied elementwise and summed with the candidate vector (Equation 10).


(10) $$h_{t}=\left(1-Z_{t}\right)\cdot h_{t-1}+\tilde{h_{t}}\cdot Z_{t}$$

where h_t_ is the hidden state output, *Z*_t_ = update gate calculated from Equation 8, h_t-1_ is the hidden state output from the previous time stamp, and $\tilde{h_{t}}$ = candidate hidden state vector calculated from Equation 9.

**Figure 5:**
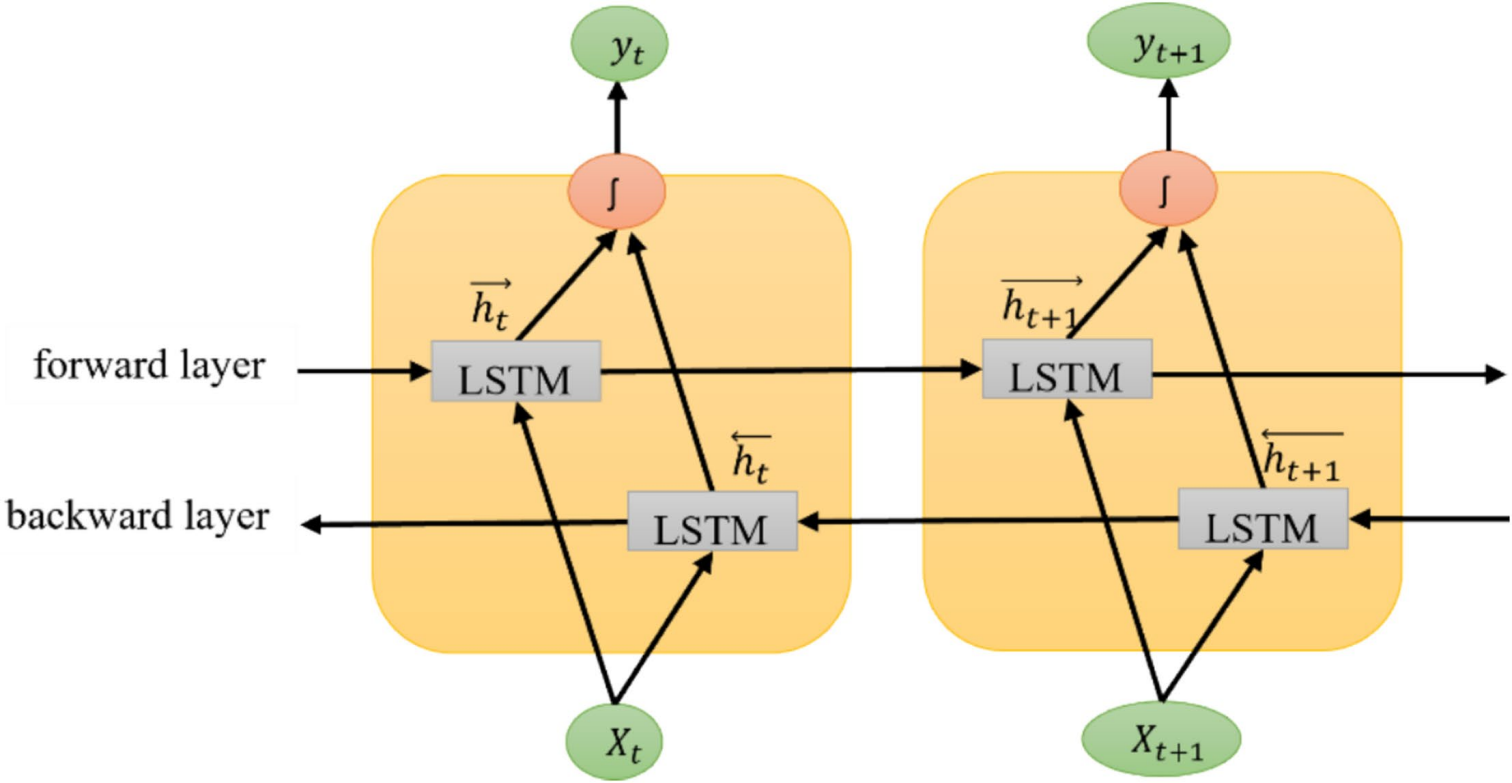
Bi-LSTM model architecture with two consecutive time frames.

The Bi-LSTM model processes the sequence in both directions of a text. One hidden layer is in the forward movement, and the other is backward. These LSTM layers are concatenated for the final output of the Bi-LSTM layer. Hence, unit 256 is doubled in this model. The return sequence parameter of LSTM is set to ‘True’ if two or more layers need to be added. The dropout parameter in the Bi-LSTM layer is set to 0.2, which helps prevent the training model from overfitting. The hidden layers are tuned from 1 to 3 in all three models. A better iteration of LSTM is the Bi-LSTM layer, which processes the sequence in forwarding and backward directions, as shown in Figure 5. The Bi-LSTM can understand the context better than the LSTM and GRU models (Li et al., 2020), as it processes input sequences in both forward and backward directions. This architecture builds upon the traditional LSTM model, enhancing its ability to capture dependencies in sequential data. In the Bi-LSTM framework, X_t_ and X_t + 1_ are the input vectors at time frame t.

While calculating the forward output sequence ($\vec{h_{t}}$), the positive sequence is used, and when calculating the backward output sequence, ($\overset{\leftarrow }{h_{t}}$), the reverse inputs are used. The output vector, y_t_, is obtained by combining the forward and backward output sequences (Equation 11).


(11) $$y_{t}=\int \left(\vec{h_{t}},\overset{\leftarrow }{h_{t}}\right)$$

where $\vec{h_{t}}$ is the forward output sequence and $\overset{\leftarrow }{h_{t}}$ is the backward output sequence. The symbol ‘ʃ’ can have different operations, such as summation, multiplication, concatenation, and average function. The default function in TensorFlow is concatenation.

The optimizer used for the compilation is adaptive moment estimation (Adam). This memory-light optimization algorithm works well with large datasets (Kingma and Jimmy, 2014). As the method label-encoder provides a sparse array of targets, the loss function uses a sparse-categorical cross-entropy.

#### 3.2.1 Hyperparameter tuning

The tuned parameters are the hidden layer and learning rate for the above models. The hidden layers are tuned from 1 to 3 in all three DL models. EarlyStopping is used in the callback application programming interface (API) of the TensorFlow model to stop overfitting the models. In this, the parameter ‘patience’ is set to 6, so the training will terminate if the validation loss function does not decrease after six epochs. Another function used is ReduceLROnPlateau. The monitoring parameter of this function is set to validity loss, patience is 3, and the minimum learning rate is 1.0*10–6. It indicates that if the loss value does not change after two epochs, the learning rate value decreases by 0.1. Thus, the new rate for the next epoch will be 0.1 times the previous rate. The most accurate model is chosen based on the accuracy of the validation set, and it is then applied to the test set.

### 3.3 Evaluation metrics

Accuracy, precision, recall, and f1-score are the metrics used to assess the classification model on the test data. For each technique, the confusion matrix is also considered. Accuracy might not be a complete metric for unbalanced data (Sturm, 2013). Therefore, precision, recall, and F1-score are also used (Grandini et al., 2020; Tharwat, 2020). The precision determines how many predicted samples are relevant (Equation 12). Recall computes how many relevant samples are predicted (Equation 13). Calculating the harmonic mean of recall and precision yields an F1-score (Equation 14). Precision is also called a positive predictive rate (PPR), and recall is known as sensitivity. Accuracy is the total sample count that was successfully predicted (Equation 15). Four performance measures are calculated using the following formulas.


(12) $$\mathrm{Precision}=\frac{\mathrm{TruePose}}{\left(\mathrm{TruePose}+\mathrm{FalsePose}\right)}$$

(13) $$\mathrm{Recall}=\;\frac{\mathrm{TruePose}}{\left(\mathrm{TruePose}+\mathrm{FalseNega}\right)}$$

(14) $$F1-\mathrm{Score}=\;\frac{2\mathrm{TruePose}}{\left(2\mathrm{TruePose}+\mathrm{FalsePose}+\mathrm{FalseNega}\right)}$$

(15) $$\mathrm{Accuracy}=\;\frac{\mathrm{TruePose}+\mathrm{TrueNega}}{\mathrm{TruePose}+\mathrm{TrueNega}+\mathrm{FalsePose}+\mathrm{FalseNega}}$$

where TruePose is a true positive, TrueNega is a true negative, FalsePose is a false positive, and FalseNega is a false negative. When the model correctly predicted the positive label, the result was considered TruePose. Similarly, if the model predicts a negative label correctly, the outcome is TrueNega. On the other hand, FalsePose is calculated based on the incorrectly predicted positive label, and FalseNega is based on the incorrectly predicted negative label.

## 4 Results

Neural networks formed the foundation of the classification models of the study, with DL techniques preferred due to the substantial volume of data involved. The experiments were conducted on a system running 64-bit Windows 10, equipped with an Intel® Core™ i7-4770K CPU at 3.50 GHz, 16 GB of RAM, and an NVIDIA GeForce GTX 1080 Ti GPU. The development environment utilized Python 3.9 and incorporated libraries such as TensorFlow 2.7 for implementing the DL models, Scikit-learn 1.0 for data preprocessing and evaluation, and PyArabic 0.6.14 for handling Arabic text processing (Abadi et al., 2016). This computational setup enabled efficient training and testing of the models, contributing to the high accuracy achieved in classifying the meters of classical Arabic poetry. The diacritics are not removed for both the full-verse and half-verse data.

### 4.1 Training and testing using full-verse data

The full-verse data are split according to 70% for training, 15% for validation, and 15% for testing. The validation accuracy according to the hidden layers is tabulated in Table 2 for the full-verse data. In addition, the number of parameters the model uses for training is specified (in millions). The trainable parameter also increases; hence, the time taken to complete the execution also increases. The training epochs are set to 60 for all the models. Callback applications such as EarlyStopping and ReduceLROnPlateau evaluate whether the model overfits. The validation loss is the parameter to check in the ReduceLROnPlateau function. If the loss value is found stable for three epochs, then the learning parameter is increased. For the EarlyStopping function, the program stops where it finds the loss value increases from the previous value or is stable for approximately six epochs. The training epochs in Table 2 show the number of epochs each model took without overfitting the data. The LSTM, GRU, and Bi-LSTM models perform better at three layers. Moreover, compared to the three models, the Bi-LSTM shows an accuracy of 97.53%.

**Table 2:** The results of increasing the layers of each model on the test accuracy of full-verse data.

Models	Hidden layers	Parameters (in millions)	Accuracy	Training Epochs	Training time (in hours)
LSTM	1	0.34	0.9720	28	89.95
	2	0.86	0.9733	26	148.17
	3	1.38	0.9737	35	286.15
GRU	1	0.26	0.9710	28	166.93
	2	0.65	0.9723	37	212.63
	3	1.05	0.9726	60	455.93
Bi-LSTM	1	0.67	0.9698	19	110.02
	2	2.24	0.9744	26	249.97
	3	3.82	0.9753	25	442.50

**Figure 6:**
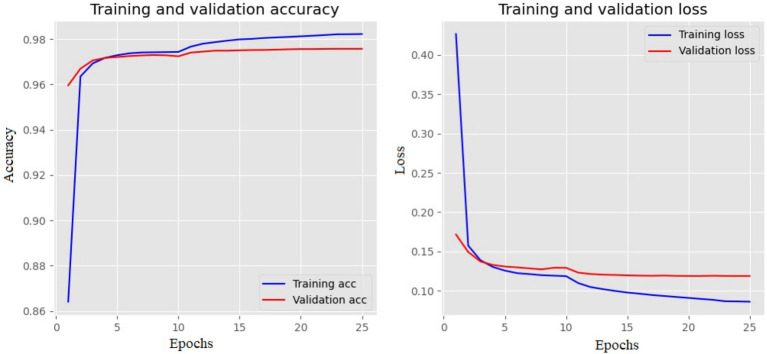
Training and validation plot of Bi-LSTM with three layers. The left side shows the accuracy, and the right shows the loss values for each epoch.

The training and validation loss and accuracy of the Bi-LSTM with three layers are depicted in Figure 6. The training loss indicates how well a DL model fits the training set. Validation loss measures the performance of the validation set. Accuracy increases as the loss value decreases.

**Figure 7:**
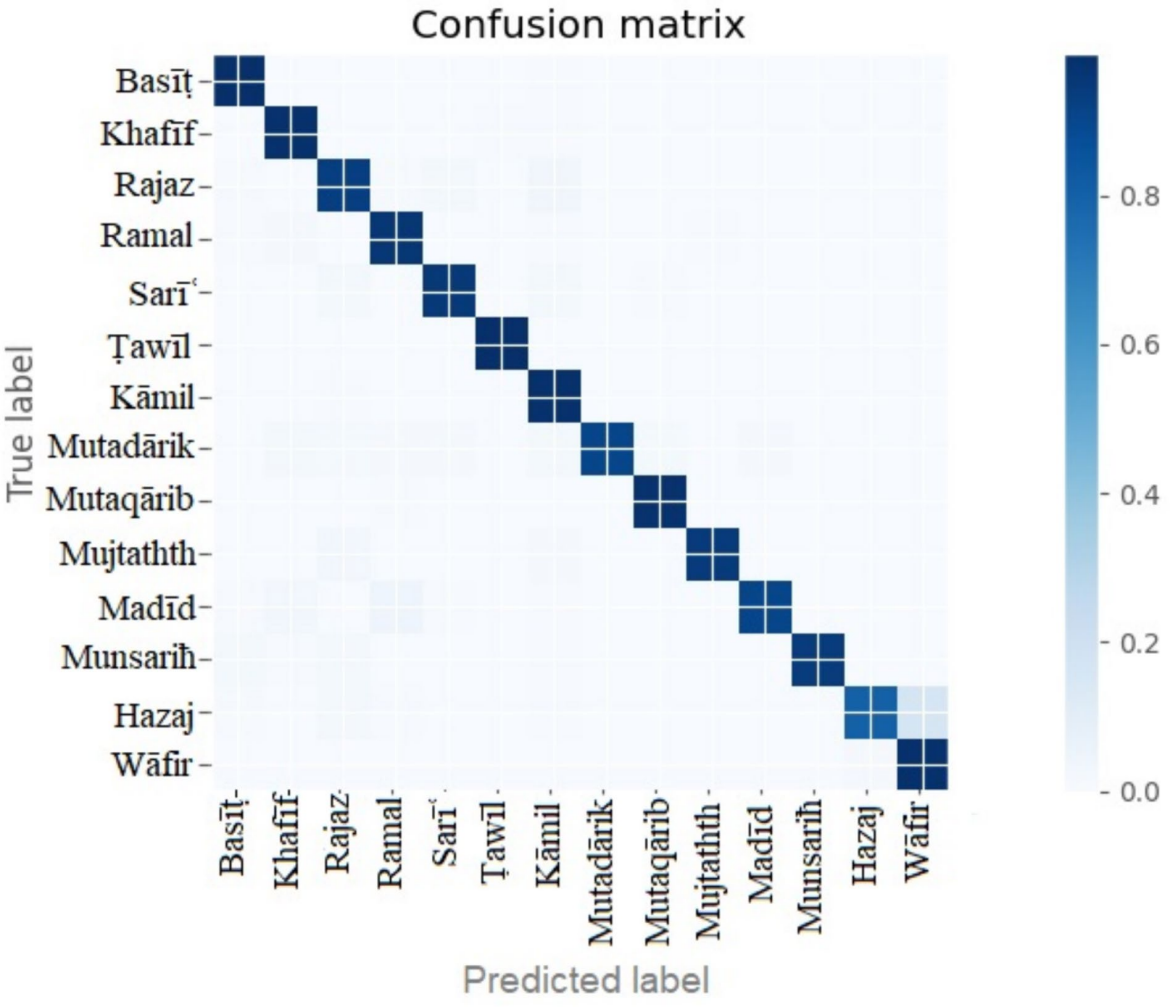
Confusion matrix of three hidden layers of the three-layer Bi-LSTM model.

The confusion matrix of the Bi-LSTM three-layer model is shown in Figure 7. The model was tested with the remaining 15% of unseen data. All the labels show good model fitting, and there was no overfitting or underfitting problem with the model performance.

The complete details of the model performance are shown in Table 3. The precision, recall, accuracy, and f1-score of each meter or label are evaluated. The basit and tawil meters show the highest accuracy of 99%. The low performance is demonstrated by the hazaj meter with 80% accuracy.

**Table 3:** Performance measure of the Bi-LSTM model with test data.

Meter	Precision	Recall	f1-score	Accuracy
Basit	0.98	0.99	0.99	0.99
Khafif	0.98	0.98	0.98	0.98
Rajaz	0.94	0.93	0.94	0.93
Ramal	0.96	0.96	0.96	0.96
Sari	0.95	0.95	0.95	0.95
Tawil	0.99	0.99	0.99	0.99
Kamil	0.97	0.98	0.98	0.98
Mutadarik	0.91	0.90	0.91	0.90
Mutaqarib	0.98	0.97	0.98	0.97
Mujtath	0.91	0.95	0.93	0.95
Madid	0.91	0.90	0.91	0.90
Munsarih	0.96	0.94	0.95	0.94
Hazaj	0.80	0.80	0.80	0.80
Wafir	0.98	0.98	0.98	0.98

### 4.2 Training and testing using half-verse

The study also implemented the model based on the half-verse data without removing diacritics. The half-verse data count is double the number of full-verse data, and the data are split into 70% training, 15% validation, and 15% testing. The hidden layers are tuned from one to three as shown in Table 4. Increasing the layers increases the parameters to train the model. In addition, the time to complete the training increases according to hidden layers. Even though the Bi-LSTM model exists in 31 epochs, it took approximately 11 h to complete the execution.

**Table 4:** The results of increasing the layers of each model on the test accuracy of half-verse data.

Models	Hidden layers	Parameters (in millions)	Accuracy	Training epochs	Training time (in hours)
LSTM	1	0.34	0.9465	34	153.23
	2	0.86	0.9494	24	166.08
	3	1.39	0.9509	28	283.33
GRU	1	0.26	0.9455	34	305.82
	2	0.65	0.9470	34	238.78
	3	1.05	0.9459	60	667.97
Bi-LSTM	1	0.67	0.9446	18	153.98
	2	2.24	0.9496	33	510.00
	3	3.82	0.9523	36	711.05

**Figure 8:**
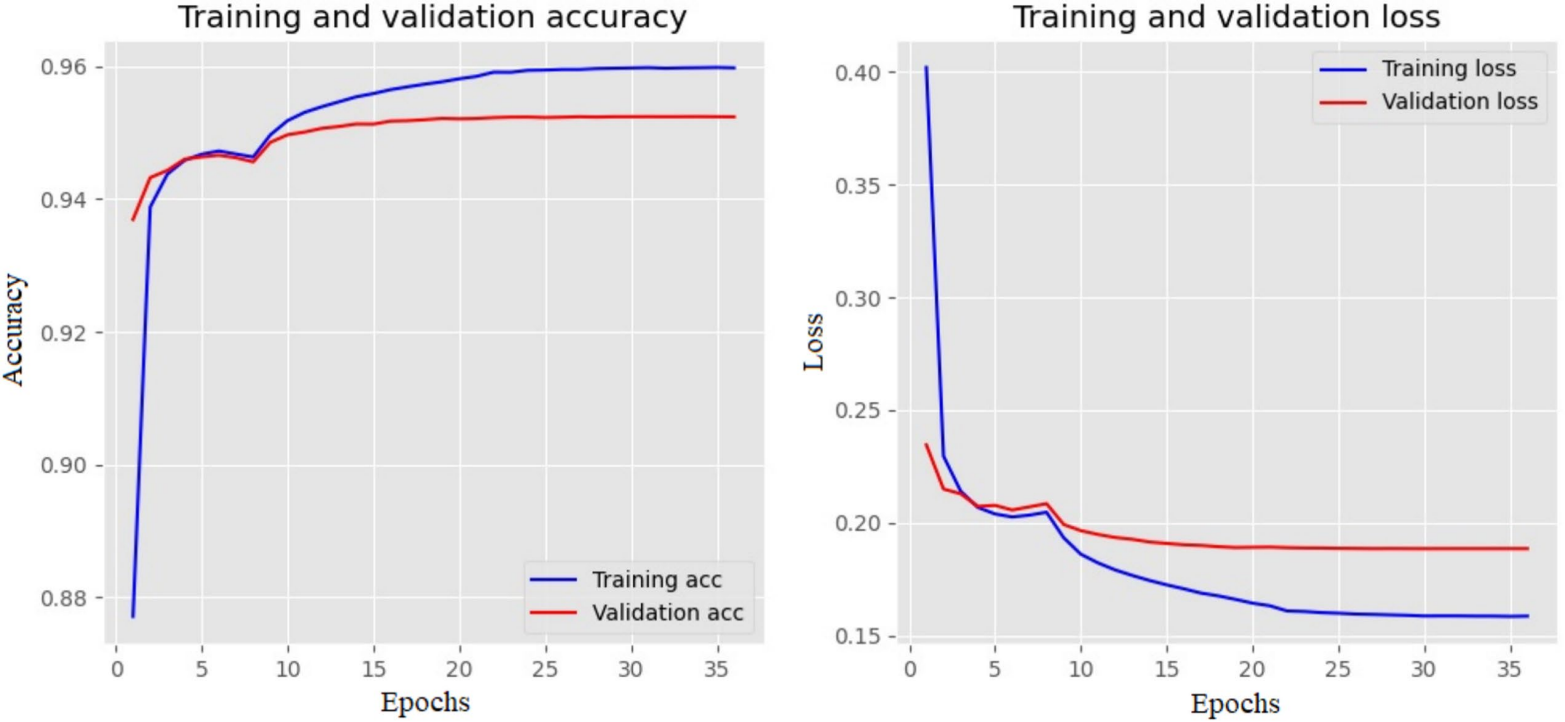
Training and validation plot of Bi-LSTM with three layers in half-verse. The left side shows the accuracy, and the right shows the loss values for each epoch.

The best model is Bi-LSTM, with 95.23% accuracy. The training and validation accuracy and loss values are shown in Figure 8. Both the loss and accuracy are inversely proportional to each other. The model exits from the iteration if the loss value is stable for six epochs.

**Figure 9:**
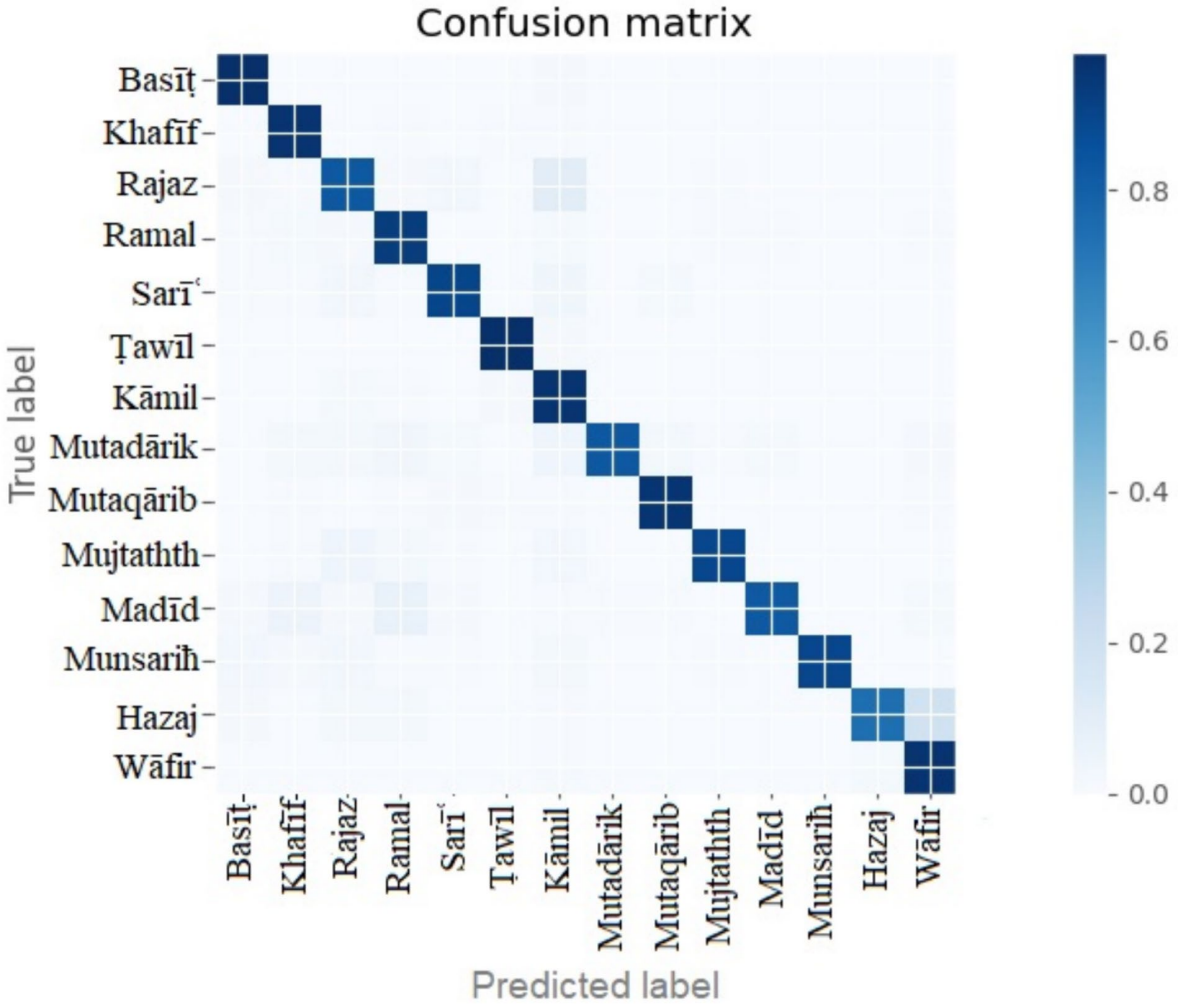
Confusion matrix of half-verse Bi-LSTM model.

The confusion matrix and the complete details of the target meters results are shown in Figure 9 and Table 5, respectively.

**Table 5:** Performance measure of the Bi-LSTM model with test data.

Meter	Precision	Recall	f1_score	Accuracy
Basit	0.98	0.98	0.98	0.98
Khafif	0.96	0.96	0.96	0.96
Rajaz	0.88	0.83	0.85	0.83
Ramal	0.92	0.93	0.92	0.93
Sari	0.91	0.90	0.90	0.90
Tawil	0.99	0.98	0.98	0.98
Kamil	0.94	0.96	0.95	0.96
Mutadarik	0.84	0.83	0.83	0.83
Mutaqarib	0.95	0.96	0.95	0.96
Mujtath	0.86	0.89	0.87	0.89
Madid	0.84	0.82	0.83	0.82
Munsarih	0.93	0.89	0.91	0.89
Hazaj	0.71	0.74	0.73	0.74
Wafir	0.97	0.96	0.97	0.96

The model shows better performance as seen in Table 5. The highest class accuracy is demonstrated by the basit and tawil meters with 98% accuracy. The lowest performance is shown by the hazaj meter, which has 74% accuracy.

## 5 Discussion

The Bi-LSTM model predicts the data better when compared with LSTM and GRU. This model’s sequence learning is in both directions, from left to right and right to left. GRU trains faster than LSTM, with fewer training parameters than LSTM (Atassi and El Azami, 2022). Few studies have been done on Arabic poetry, including the diacritization of the text data. The study by Abandah et al. (2022) showed a Bi-LSTM model with automatic diacritization. The results show a 42% improvement in the error rate of diacritization. The study by Alqasemi et al. (2021) was based on machine learning algorithms and a diacritic text. An accuracy of 96.34% was achieved using support vector machines (SVM). Another study by Al-shathry et al. (2024) employed a balanced dataset by randomly choosing 1,000 poem verses for each meter. Their study achieved 98.6% accuracy, but 90% precision, recall, and f1-score value with the Bi-GRU model.

The proposed study can be compared with the studies by Abandah et al. (2020) and Al-shaibani et al. (2020). With five hidden layers, Al-shaibani et al. (2020) reached an accuracy of 94.32% with the bi-directional GRU (Bi-GRU) model and 14 target meters. The model also attains 88.8% accuracy for half-verse data. With four hidden layers, the Bi-LSTM model by Abandah et al. (2020) achieved an accuracy of 97% without removing diacritics and 97.27% with removed diacritics. They use 16 meters as target classes. The study carried out by Yousef et al. (2019) used seven hidden layers for the Bi-LSTM model and achieved an accuracy of 96.38%. In the proposed research, the number of verses is much higher than in the study done by Al-shaibani et al. (2020). In addition, the number of hidden layers is less than in all three studies. The comparison of Arabic meter studies is mentioned in Table 6.

**Table 6:** Comparison between related studies in literature and the proposed study.

Reference	Technique used—number of hidden layers	Dataset size	Accuracy	F1-score
Al-shaibani et al. (2020)	Bi-GRU-5	55,400 verses	94.32% (full-verse), 88.80% (half-verse)	-
Abandah et al. (2020)	Bi-LSTM-4	1,657,003 verses	97.27% (full-verse)	0.97 (full-verse)
Yousef et al. (2019)	Bi-LSTM-7	1,722,321 verses	96.38% (full-verse)	-
The proposed work	Bi-LSTM-3	1,646,771 verses	97.53% (full-verse), 95.23% (half-verse)	0.98 (full-verse), 0.95 (half-verse)

The studies (Abandah et al., 2020; Yousef et al., 2019) employed the identical dataset as the proposed study, although it documented varying verse counts. This suggests that although the dataset is uniform, discrepancies in verse counts may influence model efficacy. The models employed in the compared research, Bi-LSTM with four and seven layers, attained competitive accuracy rates; nevertheless, the proposed Bi-LSTM model with three layers surpassed them across all criteria. The study by Al-shaibani et al. (2020) utilized a distinct dataset; however, it similarly extracted poems from the ‘Aldiwan’ website. The Bi-GRU model employed in the mentioned study (Al-shaibani et al., 2020) shows worse performance measures relative to the proposed study findings. The variations in dataset construction and model design certainly led to the noted performance variances.

In the proposed study, the Bi-LSTM model with three hidden layers performs better than one or two hidden layers without removing diacritical text. In addition, it better predicts than the LSTM and GRU models for both full-verse and half-verse data. LSTM cannot use future tokens nor can local contextual information be extracted. This problem can be resolved using Bi-LSTM, which learns the sequence in forward and backward directions. GRUs are faster to train than the LSTM model but lack the output gate. The model achieved an accuracy of 97.53% for the full-verse data and 95.23% for the half-verse data.

The results of the study suggest that the number of hidden layers significantly impacts the performance of the Arabic meter classification model using Bi-LSTM. The study achieved better accuracy in Arabic meter classification using Bi-LSTM models with three hidden layers than previous studies that used Bi-LSTM models with four and seven hidden layers. It suggests that increasing the number of hidden layers beyond a certain point may not always lead to better performance and that optimizing the number of hidden layers can be a crucial factor in achieving high accuracy.

A few baseline ML models were utilized in this study to evaluate their performance in comparison with the DL architectures used for the Arabic poetry meters’ classification. It includes a decision tree (DT), random forest (RF), k-nearest neighbors (KNN), and extra tree (ET) classifier. These classifiers serve as effective benchmarks for evaluating the performance of more complex models. The DT model yielded an accuracy of 46% with an F1-score of 0.30, and KNN achieved 30% with a 0.20 F1-score, while the ensemble models RF and ET achieved 58 and 53% accuracy as well as 0.50 and 0.56 F1-score values, respectively.

The comparison with baseline models underscores the efficacy of the DL methodologies utilized in the proposed study. Although baseline models serve as a valuable foundation, advanced models (Bi-LSTM) exhibit significant enhancements in accuracy and overall performance. This highlights the need to employ DL methodologies for intricate tasks such as Arabic poetry meter classification, where conventional models might struggle to grasp the complex nature of the data.

### 5.1 Practical implications

The findings of the proposed study on the categorization of Arabic poetry meter using DL models have substantial practical applications in several fields. This research enhances NLP, text analytics, and cultural heritage preservation by attaining high accuracy in the classification of full and half verses of Arabic poetry.

- Accurate classification of Arabic poetry meters helps preserve Arabic literary legacy. Automating the study of poetic structures helps scholars and cultural organizations to better classify historical data, therefore guaranteeing their availability for the next generations.
- The proposed DL system may be included in learning environments to support academics and students in comprehending Arabic poetry. By giving instantaneous feedback and poetic work analysis, interactive technologies that use meter classification can improve learning opportunities and help increase the importance of Arabic literature.
- Other kinds of Arabic literature can be examined using the approach developed in this study. Adapting the models to several literary genres allows scholars to investigate structures and patterns that define distinct kinds of Arabic literature, therefore enhancing the knowledge of the literary scene of the language.
- Using the knowledge acquired from the proposed study, NLP practitioners may increase the performance of the model in processing the Arabic text, therefore enhancing its applicability in fields such as social media analysis and automatic content development.

## 6 Conclusion

This study presents a significant advancement in the automatic classification of classical Arabic poetry meters using deep learning techniques. By utilizing a substantial dataset of 1,646,771 verses without removing diacritics, the Bi-LSTM models with three hidden layers were developed and evaluated. The Bi-LSTM model outperformed traditional LSTM and GRU models, achieving an accuracy of 97.53% on full-verse data and 95.23% on half-verse data. These results surpass those of previous studies that employed models with more hidden layers or smaller datasets.

The superior performance of the Bi-LSTM model underscores its effectiveness in capturing the complex rhythmic and phonetic patterns inherent in classical Arabic poetry. The ability of Bi-LSTM to process sequences in both forward and backward directions allows for a more comprehensive understanding of the linguistic structures involved. Importantly, retaining diacritics in the text preserved essential phonetic information, which proved crucial for accurate meter classification.

The findings of the study make a substantial contribution to computational linguistics and natural language processing, particularly in the context of Arabic language studies. The high accuracy achieved demonstrates the potential of the model for practical applications, such as automated literary analysis and educational tools that enhance the study and appreciation of Arabic poetry. This study also aligns with the Sustainable Development Goals by promoting quality education and fostering innovation in language technology.

### 6.1 Limitations and future studies

The proposed study performs better with half-verse and full-verse Arabic poems. It indicates that although the average accuracy is elevated, some classes, especially those corresponding to meters with fewer verses, demonstrate diminished precision and recall. Future studies must concentrate on these underrepresented categories to enhance their classification efficacy. This can be accomplished using specific data augmentation procedures, such as the generation of synthetic examples of certain meters or the application of oversampling techniques to equilibrate the dataset.

Although several DL models were evaluated, their hyperparameters, such as optimizers and the number of units in layers, were not extensively tuned. Hyperparameter selection may greatly affect the model’s performance. Future studies should consider using methodical hyperparameter tuning strategies to improve model performance. Another scope of future studies is to investigate the influence of other linguistic attributes on meter classification. It includes semantic and syntactic structure analysis.

## Data Availability

Publicly available datasets were analyzed in this study. This data can be found at: https://arxiv.org/abs/1905.05700.
